# Intraarticular senescent chondrocytes impair the cartilage regeneration capacity of mesenchymal stem cells

**DOI:** 10.1186/s13287-019-1193-1

**Published:** 2019-03-12

**Authors:** Xu Cao, Pan Luo, Junjie Huang, Chi Liang, Jinshen He, Zili Wang, Dongyong Shan, Cheng Peng, Song Wu

**Affiliations:** 1 Department of Orthopaedics of the 3rd Xiangya Hospital, Central South University, Changsha, 410013 China; 2 Department of Oncology of the 3rd Xiangya Hospital, Central South University, Changsha, China; 3 Department of Burns and Plastic Surgery of the 3rd Xiangya Hospital, Central South University, Changsha, 410013 China

**Keywords:** Senescent chondrocytes (SnChos), Bone marrow mesenchymal stem cells (BMSCs), Intra-articular senescent microenvironment (IASM), Osteoarthritis (OA), Cartilage repair

## Abstract

**Background:**

Senescent cells exert a significant influence over their surrounding cellular environment. Senescent chondrocytes (SnChos) were found to be accumulated in degenerated cartilage present in joints affected by osteoarthritis. The influence of SnChos on exogenously transplanted stem cells has yet to be reported.

**Methods:**

In this study, we evaluated the interactions between SnChos and bone marrow mesenchymal stem cells (BMSCs) when co-cultured as well as in the intra-articular senescent microenvironment (IASM). The effect of IASM on cartilage regeneration was also assessed.

**Results:**

It was found that a small fraction of SnChos induced BMSC cellular senescence and apoptosis. SnChos also inhibited proliferation, facilitated stemness, and suppressed chondrogenic differentiation of BMSCs. BMSCs induced the apoptosis of SnChos, reduced the proportion of SnChos, stimulated SnChos proliferation, and revealed a bidirectional effect on SnChos inflammaging. IASM significantly suppressed the survival, proliferation, and appropriate differentiation of grafted BMSCs in vivo, all of which impaired cartilage regeneration. Anti-senescence agent ABT-263 was able to partly rescue the cells from the negative effects of SnChos.

**Conclusions:**

The SnChos and BMSCs interacted with each other at cellular senescence, apoptosis, proliferation, differentiation, and cell functions. This interaction impaired the cartilage repair of MSCs. Anti-senescence agent provided a possible solution for this impairment.

**Electronic supplementary material:**

The online version of this article (10.1186/s13287-019-1193-1) contains supplementary material, which is available to authorized users.

## Background

Stem cell therapy is currently recognized as a promising strategy in osteoarthritis (OA) treatment. However, clinical trials investigating its effects resulted in a broad range of outcomes [1]. While generally positive therapeutic effects were reported, none of the trials have provided results supporting the ability of stem cell therapy to provide adequate symptom relief or its ability to prevent OA progression.

The incidence of OA increases with age. It is a degenerative disease involving cellular senescence, a concept first described by Hayflick and Moorhead [2]. In a joint afflicted by OA, there is an accumulation of senescent cells (SnCs), and its quantity has been positively correlated with OA severity [3]. Recent studies have demonstrated that these SnCs promote a conducive microenvironment that supports the progression of OA [4, 5]. This discovery led to the concept of novel mechanism of treatment of OA. In fact, studies on several other age-related diseases have begun focusing on the impact of different SnCs on the surrounding cells and microenvironment. SnCs are thought to exert their influence over other cells by exhibiting a senescence-associated secretory phenotype (SASP) [6]. The interactions between SnCs and stem cells have particularly been investigated, given the prominent role of regenerative medicine in age-related diseases [7]. These interactions have been found to be significant in the context of affecting tissue regeneration in age-related diseases. However, this interaction in OA-afflicted joints still remains unknown.

While OA is recognized as a disease that involves the entire whole joint, it is primarily a disease of the cartilage. In 2002, Price et al. was the first to observe the accumulation of senescent chondrocytes (SnChos) in degenerative OA cartilage [8]. However, no significance was attached to the presence of these SnChos. Cartilage repair is the key objective of stem cell therapy for OA. It remains a challenge for stem cells to generate long-lasting hyaline cartilage in joints afflicted by OA. After transplantation, stem cells are faced with three critical obstacles in cartilage repair, including the decline in cell quantity that occurs as a result of cell apoptosis and proliferation restriction, the insufficient capacities for the cells to synthesize matrix proteins, and the difficulty in inducing and maintaining the chondrogenic phenotype [9]. The microenvironment in joint cavity has critical effects on stem cell functions [10]. SnChos are likely to impact the viability, proliferation, differentiation, and matrix synthesis of stem cells, thus impairing stem cell ability to repair cartilage. On the other hand, grafted stem cells may also affect SnChos. These interactions, which have yet to be clearly illustrated, are crucial in the successful application of regenerative medicine. Therefore, in this study, we investigated the in vitro and in vivo interactions between bone marrow mesenchymal stem cells (BMSCs) and SnChos in order to determine their impact on cartilage regeneration.

## Materials and methods

### BMSC isolation and culture

The BMSCs were obtained using methods described in our previous study that involves identification of the stem cells via cell morphology, surface biomarkers, and multiple differentiation capacities (Additional file 1: Figure S1) [11]. In brief, MSCs were first isolated from the bone marrow (BM) of the tibia and femur of 12 Sprague-Dawley (SD) rats (3 weeks old, male). Cells were pooled together and cultured in DMEM (Gibco, USA) supplemented with 10% FBS (Gibco, USA) at 37 °C. The medium was replaced every 3 days, and cells were cultured until 80% confluence was achieved. BMSCs in passage 3 were used for co-culture and transplantation.

### Normal chondrocytes (Chos) isolation and culture

Chos were obtained and identified (Additional file 1: Figure S1) using methods described in our previous study [12]. In brief, the articular cartilage of the tibial plateau and distal femur of 12 SD rats (3 weeks old, male) were harvested and digested with 1 mg/ml type II collagenase (Sigma, USA) at 37 °C. Chos were pooled, identified using type II collagen ICC, and cultured in DMEM (Gibco, USA) supplemented with 10% FBS (Gibco, USA) at 37 °C. Chos in passage 3 were used for co-culture or further senescence induction.

### The induction of senescent chondrocytes (SnChos) and intra-articular senescent microenvironment (IASM)

Chos in passage 3 that achieved approximately 70% confluence were exposed to ionizing radiation (IR) at a dose rate of 1.080 Gy/min for 10 min using a J.L. Shepherd Model Mark I ^137^Cesium γ-irradiator (J.L. Shepherd, USA). Chos were cultured for another 10 days after IR in order to fully induce senescence prior to being utilized for co-culture. For the anti-senescence group, 1 μM ABT-263 (Selleck, USA) was added into the culture medium for 3 days prior to co-culture. The SnChos was confirmed through SA-β-Gal staining. For IASM induction, the knees of 1-month SD rats were exposed to IR at a dose rate of 1.080 Gy/min for 10 min. Other body regions of rats were prevented from IR exposure. Rats were free to move for 3 months post-IR in order to achieve full senescence before surgery and MSC transplantation. For the antisenescence group, 0.1 ml 1 μM ABT-263 (Selleck, USA) was injected intra-articularly once a week during the last month of 3 months above. Senescent cartilage was confirmed through detection of p16^Ink4a^ and p21^Cip1^ expressions.

### The co-culture of BMSCs with SnChos

A co-culture system was established using six-well Transwell plates (Corning, USA). 3 × 10^5^ BMSCs were cultured in the upper compartments and 3 × 10^5^ SnChos, Chos, or SnChos that had been pretreated by ABT-263 were cultured in the lower compartments in DMEM with 10% FBS. Chondrogenic differentiation medium (Gibco, USA) was used to culture for MSC stemness and to evaluate differentiation. BMSCs and SnChos alone were cultured as the control. Co-cultures were maintained for 7 and 21 days before evaluation. Cells were passaged every 7 days during the 21-day co-culture. Co-cultures were conducted in technical triplicate for each assay and five random field of each well were selected for evaluation.

### Establishment of rat cartilage defect (CD) model

Untreated, IR-pretreated, and IR/ABT-pretreated rats (16 rats for each group) were evaluated for cartilage regeneration. A cartilage defect (CD) with 2 mm in diameter, 3.5 mm in depth, was made at the middle of right femoral trochlea using a punch. The defect was filled with BMSCs embedded in a low gelling temperature agarose gel scaffold (Sigma, USA) at a density of 2 × 10^5^ cells/ml. Rats were sacrificed at 1 week (8 rats/group) for BMSC viability, apoptosis, proliferation, and senescence evaluation, at 6 weeks (5 rats/group) for Col II, Agg, and safranin O staining and at 12 weeks (3 rats/group) to evaluate for cartilage repair.

### EdU incorporation assay

Proliferating cells were determined by the Click-iT Plus EdU Alexa Fluor 488 Imaging Kit (Invitrogen, USA). Briefly, cells were incubated with 10 μM EdU for 3 h before EdU staining. Cell nucleus were stained with Hoechst 33342 (Invitrogen, USA) at 5 μg/mL for 30 min. The cells were counted in five random fields per well. The percentage of EdU-positive cells was calculated using Image-Pro Plus version 6.0 for Windows (Media Cybernetics, USA). For MSC grafts evaluation, 0.1 ml 10 μM EdU was injected intra-articularly at 7 days after transplantation. One day later, rats were sacrificed and the femoral trochlea were harvested. The frozen sections of BMSC grafts were evaluated using the EdU Kit and stained with Hoechst 33342 at 5 μg/mL for 30 min. Each frozen section was counted in five random fields.

### Immunostaining for caspase-3

Culture medium was removed, and cells were washed thrice with PBS. After 0.1% Triton X-100 and 3% H_2_O_2_ treatment, cells were incubated in rabbit polyclonal anti-Caspase-3 antibody (ab13847, Abcam, UK) at 4 °C overnight and anti-rabbit universal immunoassay kit (GeneTech, China) at 37 °C for 30 min. The cells were counted in five random fields per well. For BMSCs grafts evaluation, rats were sacrificed at 7 days after transplantation and the femoral trochlea were harvested. Frozen sections were stained by anti-Caspase-3 antibody (ab13847, Abcam, UK) at 4 °C overnight, and anti-rabbit immunoassay kit (GeneTech, China) at 37 °C for 1 h. Each frozen section was counted in five random fields.

### Real-time quantitative polymerase chain reaction (RT-qPCR)

Total RNA of cells was extracted using the RNA simple Total RNA Kit (DP419, TIANGEN, China). Reverse transcription reactions were performed using RevertAid First Strand cDNA Synthesis Kit (K1622, ThermoFisher, USA). RT-qPCR reactions were conducted using SuperReal PreMix Plus (SYBR Green, FP205-02, TIANGEN, China). The initial denaturation was 95 °C for 15 min, and 45 cycles of denaturation at 95 °C for 10 s, annealing at 60 °C for 20 s and extension at 72 °C for 30 s. The primer sequences are shown in Table 1. The transcriptional levels of genes were normalized to β-actin and calculated using the lg^2–△△Ct^.

**Table 1:** Gene-primer sequences for RT-qPCR

Name	Forward primer	Reverse Primer
Bax	CATGGAGCTGCAGAGGATGA	GAGGAAGTCCAGTGTCCAGC
Bcl-2	GAACTGGGGGAGGATTGTGG	ACAAAGGCATCCCAGCCTC
CDKN2A	CGTACCCCGATACAGGTGATG	ATACCGCAAATACCGCACGA
CDKN1A	AGCAGTTGAGCCGCGATTG	ACCCAGGGCTCAGGTAGATCTTG
IL-6	CTCCGCAAGAGACTTCCAGC	TCTGACAGTGCATCATCGCT
MMP1	ATGAGACGTGGACCGACAAC	TGAGTGAGTCCAAGGGAGTG
MMP13	GCAGCTCCAAAGGCTACAAC	TCTGGTGTTTTGGGGTGCTT
Sox9	CTGAAGGGCTACGACTGGAC	CTCTCGTTCAGCAGTCTCCA
IL-1β	GGCTTCCTTGTGCAAGTGTC	TGTCGAGATGCTGCTGTGAG
COLL II	GCCAGGATGCCCGAAAATTAG	CATGACTCCCATCTGGGCAC
Nanog	TTGGGCTGACATGAGCGTG	GAGGAGAGGCAGTCTCTGTG
Oct-4	CACGAGTGGAGAGCAACTCG	AGATGGTTGTCTGGCTGAAC

### Senescence-associated β-galactosidase staining

After being fixed by 4% paraformaldehyde for 15 min and washed thrice with PBS, cells were stained by Senescence Detection Kit (K320-250, BioVision, USA) according to the manufacturer’s protocol. The cells were counted in five random fields per well. Cell nucleus was stained by DAPI (Boster, China). For BMSC grafts’ evaluation, rats were sacrificed at 7 days after transplantation and the femoral trochlea were harvested. Frozen sections were stained by Senescence Detection Kit according to the manufacturer’s protocol. DAPI was used for nucleus staining. Cells were counted in five random fields per section.

### Western blotting for Col II and Agg

The Col II and Agg production was evaluated by WB at 21 days of co-culture. Briefly, protein extracts were separated by SDS-PAGE and blotted onto polyvinylidene fluoride membranes (Immobilon P, USA). The membranes were incubated with anti-Collagen II (ab34712, Abcam, UK) and anti-Aggrecan (MA3-16888, Thermo Fisher, USA) at 4 °C overnight and with horseradish peroxidase-conjugated secondary antibody (Cell Signaling Technology, USA) at 37 °C for 1 h. Protein bands were imaged by ChemiDoc XRS Plus luminescent image analyzers (Bio-Rad, Hercules, CA, USA). The protein levels were normalized to GAPDH.

### AM/PI staining for BMSCs grafts

0.1 ml 2 μmol/L Calcein-AM and 4 μmol/L PI (YEASEN, China) were injected intra-articularly at 7 days after transplantation. One day later, rats were sacrificed and the femoral trochlea were harvested. The frozen sections of BMSCs grafts were evaluated for five random fields per section by fluorescent microscope (Olympus, Japan).

### Histological and immunohistochemical evaluation

At 6 and 12 weeks after transplantation, rats were sacrificed and the femoral trochlea were harvested. Frozen sections were stained by IHC using anti-Collagen II (ab34712, Abcam, UK), anti-Aggrecan (MA3-16888, Thermo Fisher, USA) and anti-Collagen I (380760, ZenBio, CHN) at 4 °C overnight, and horseradish peroxidase-conjugated secondary antibody (Cell Signaling Technology, USA) at 37 °C for 1 h. Sections were also stained for histological evaluation using safranin O-fast green staining. The residual immature area (RIA) and residual gel area (RGA) at 6 weeks were calculated by ImageJ V1.8.0 (National Institutes of Health, Bethesda, MD, USA). The cartilage regeneration at 3 months was evaluated by the Pineda score [13].

### Statistical analysis

Values are expressed as the mean ± SD in the text and figures unless otherwise noted. Statistical significance was determined by Student’s *t* test or analysis of variance (ANOVA) using SPSS 17.0 (SPSS, Inc., Chicago, IL, USA). *P* < 0.05 was considered to be statistically significant.

## Results

### Confirmation of senescence induction

**Fig. 1:**
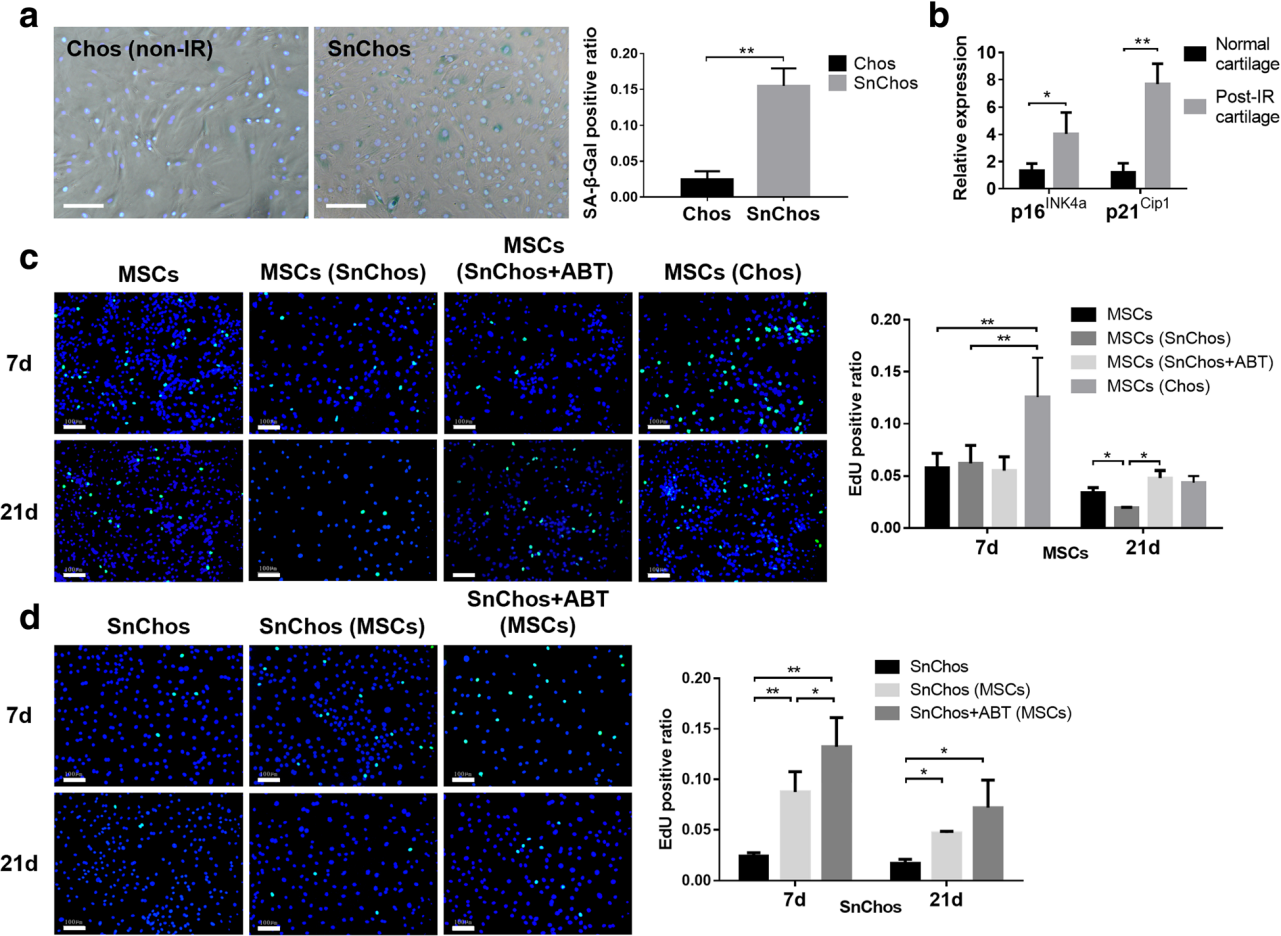
The confirmation of senescence induction and proliferation of cells in co-culture. **a** The staining for SA-β-Gal (green) in non-IR Chos (left) and SnChos (right) 10 days after IR exposure. The ratio of SA-β-Gal-positive cells was calculated (right, *n* = 3). **b** The expression level of p16^Ink4a^ and p21^Cip1^ in cartilage from non-IR rat joints and post-IR rat joints (n = 3). **c**, **d** The EdU staining (green) in proliferating BMSCs (**c**) and SnChos (**d**) in co-culture at 7 and 21 days. Nucleus were stained by Hoechst 33342 (blue). Bars = 100 μm. The ratio of EdU-positive cells was calculated (right, *n* = 3). *P < 0.05, **P < 0.01

Ten days after IR exposure, SnChos exhibited evident senescent phenotype. Fifteen percent SnChos were positively stained by SA-β-Gal. There were only 3% non-IR chondrocytes revealed SA-β-Gal staining (Fig. 1a). Three months after IR exposure, the expression of senescent markers p16^Ink4a^ and p21^Cip1^ elevated significantly in cartilage of knee joint (Fig. 1b).

### Cell proliferation and apoptosis

After 7 days of co-culture, normal chondrocytes (Chos) stimulated the proliferation of BMSCs. This intrinsic stimulation was completely counteracted by the existence of SnChos. At 21 days, the augmentative effect of Chos disappeared. SnChos was found to inhibit MSC proliferation. This inhibition was eliminated by pre-treatment with antisenescence agent ABT-263 (Fig. 1c).

SnChos exhibited an obvious arrest in cell cycle. At 7 days, BMSCs significantly improved this senescence-related suppression of proliferation. At 21 days, the enhancing effects of BMSCs declined. ABT pretreatment further facilitated the proliferation of SnChos co-cultured with BMSCs (Fig. 1d).

**Fig. 2:**
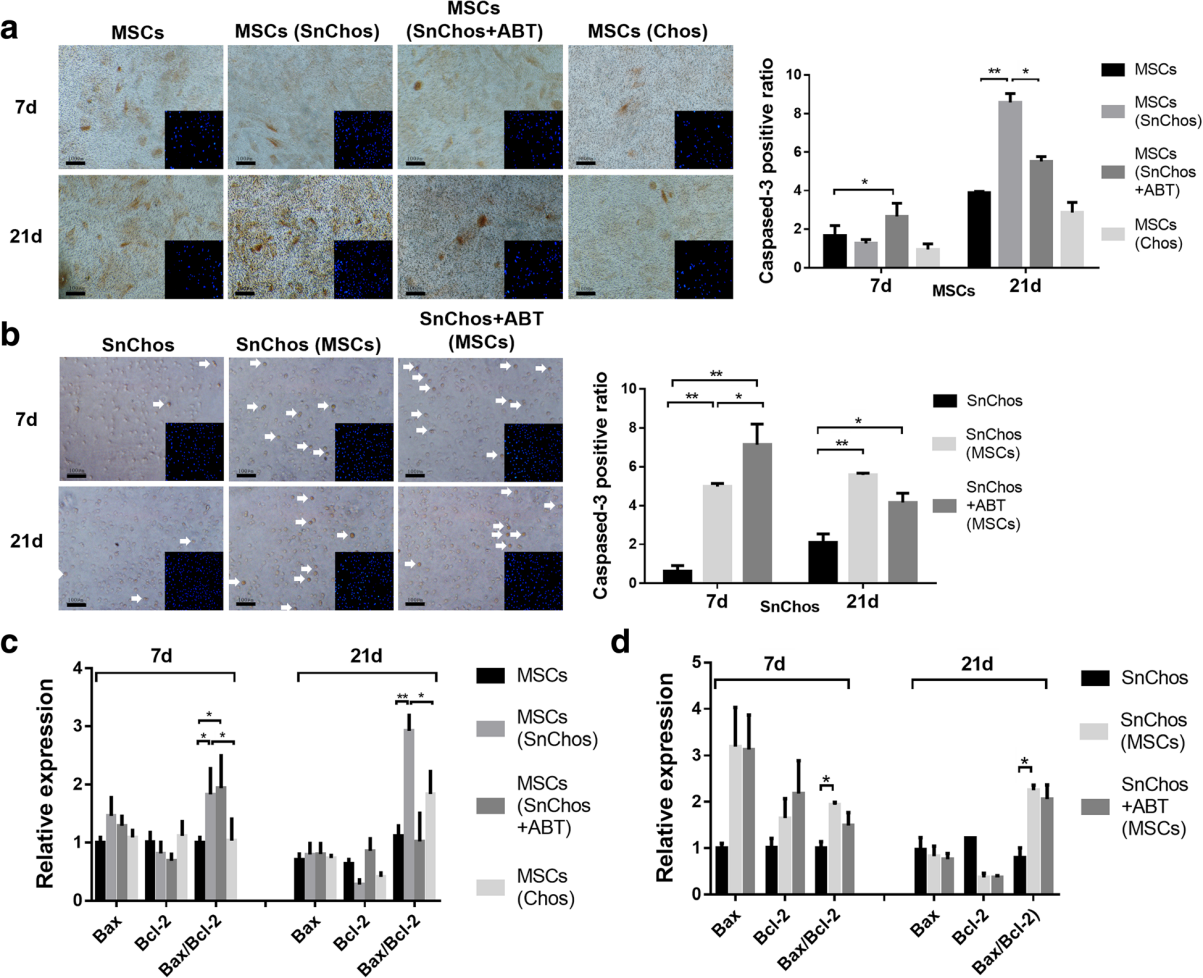
The apoptosis of cells in co-culture. **a**, **b** The immunocytochemistry staining for caspase-3 (brown) in apoptotic BMSCs (**a**) and SnChos (**b**, arrows) in co-culture at 7 and 21 days. Bars = 100 μm. The ratio of caspase-3-positive cells was calculated (right, *n* = 3). **c**, **d** The expression level of Bax and Bcl-2 in BMSCs (**c**) and SnChos (**d**) in co-culture at 7 and 21 days (*n* = 3). The BMSCs (7d) or SnChos (7d) were used as control. *P < 0.05, **P < 0.01002E

At 7 days, neither SnChos nor Chos significantly affected BMSC apoptosis. However, at 21 days, both caspase-3 staining and Bax/Bcl-2 expression ratio indicated that SnChos significantly promoted MSC apoptosis. ABT-263 alleviated this apoptosis induction (Fig. 2a, c).

Caspase-3 and Bax/Bcl-2 expression ratios revealed that BMSCs also conferred a remarkable apoptosis promotive effect to SnChos within 21 days of co-culture (Fig. 2b, d). ABT further promoted the apoptosis of co-cultured SnChos at 7 days, but had no effect at 21 days (Fig. 2b).

### Cellular senescence and inflammaging

**Fig. 3:**
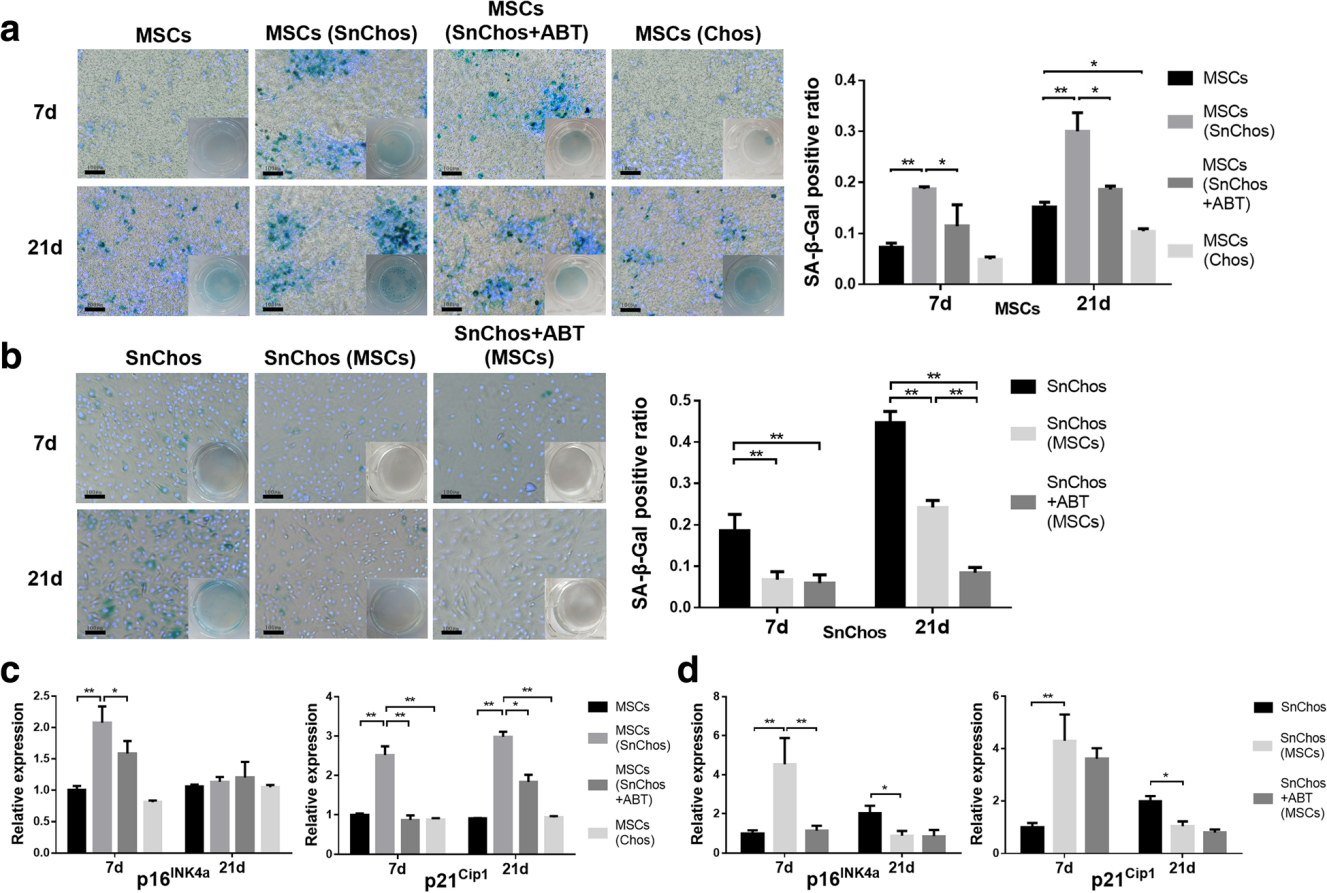
The senescence of cells in co-culture. **a**, **b** The staining for SA-β-Gal (green) in senescent BMSCs (**a**) and SnChos (**b**) in co-culture at 7 and 21 days. Bars = 100 μm. The ratio of SA-β-Gal positive cells was calculated (right, *n* = 3). **c**, **d** The expression level of p16^Ink4a^ and p21^Cip1^ in BMSCs (**c**) and SnChos (**d**) in co-culture at 7 days and 21 days (*n* = 3). The MSCs (7d) or SnChos (7d) were used as control. *P < 0.05, **P < 0.01

Overall, prolonged culture resulted in cellular senescence. Chos had a mild anti-senescence effect on BMSCs, while SnChos markedly promoted MSCs senescence. Up to 30% of BMSCs were induced by SnChos to express SA-β-Gal at 21 days. ABT alleviated this effect to 19% (Fig. 3a). Likewise, SnChos induced BMSCs to express senescence markers p16^Ink4a^ and p21^Cip1^ in an asynchronized manner (Fig. 3c). In contrast, BMSCs exhibited a significant antisenescence effect on SnChos, as revealed by a lower proportion of SA-β-Gal-positive cells in the SnChos group during 21 days of co-culture. At 21 days, ABT further reduced the SA-β-Gal-positive SnChos (Fig. 3b). Meanwhile, BMSCs exhibited an interesting pattern of effects on SnChos. In the 7 days co-culture, we noted a transient increase of p16^Ink4a^ and p21^Cip1^ in SnChos, in contrast to the prolonged culture which exhibited an inhibitory effect (Fig. 3d).

**Fig. 4:**
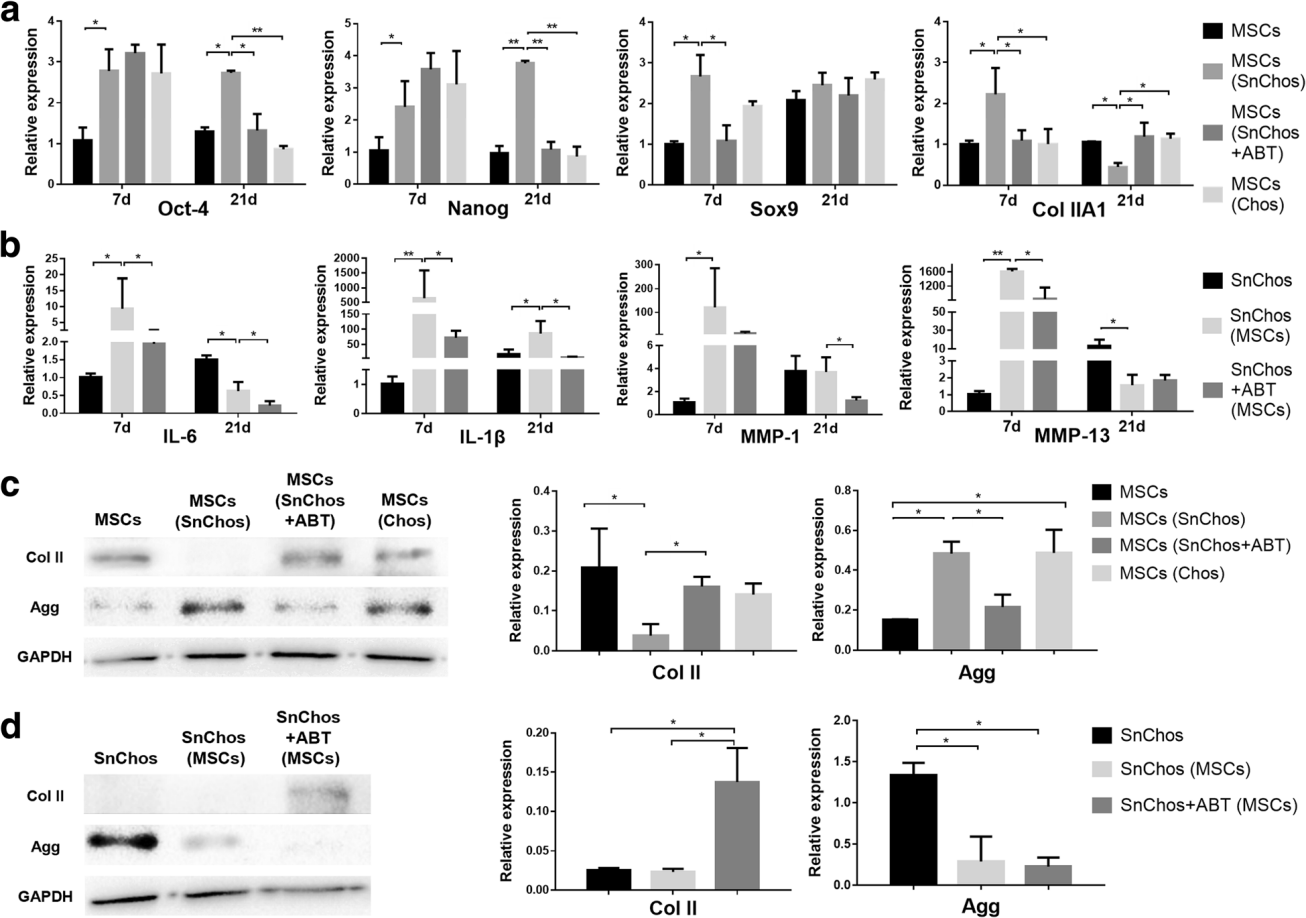
The inflammation, differentiation, and chondrogenesis. **a**, **b** The expression level of stemness and differentiation markers in BMSCs (**a**) and inflammatory factors in SnChos (**b**) in co-culture at 7 and 21 days (*n* = 3). The MSCs (7 days) or SnChos (7 days) were used as control. **c**, **d** The generation of cartilage matrix protein Col II and Agg by BMSCs (**c**) and SnChos (**d**) in co-culture at 21 days (*n* = 3). The protein levels were normalized to GAPDH. *P < 0.05, **P < 0.01

The variation of SASP factors of SnChos is similar to that of senescence markers above. In brief, MSCs induced a transient sharp increase of IL-6, IL-1β, MMP-1, and MMP-13 expressions by SnChos at 7 days, but inhibited the IL-6 and MMP-13 at 21 days. ABT pretreatment revealed an inhibitory effect on these SASP factors expression of SnChos co-cultured with BMSCs (Fig. 4b).

### MSCs differentiation and chondrogenesis

At 7 days, Chos transitorily facilitated the stemness of BMSCs, represented by expressions of Oct-4 and Nanog. In contrast, SnChos persistently promoted BMSCs stemness throughout 21 days of co-culture. Meanwhile, SnChos stimulated the expression of chondrogenic markers Sox9 and Col II by MSCs at 7 days. This stimulative effect disappeared and evolved into a suppressive effect at 21 days. These effects of SnChos were eliminated by ABT-263 pretreatment (Fig. 4a).

Chondrogenesis was evaluated by Agg and Col II at 21 days. SnChos revealed marked stimulative and inhibitory effect to Agg and Col II production of BMSCs respectively. ABT pretreatment attenuated these effects. Chos stimulated BMSC Agg production and had no effect on Col II (Fig. 4c). In return, BMSCs suppressed the Agg production of SnChos and had no effect on Col II. In contrast, ABT pretreatment improved Col II production of SnChos while had no effect on Agg (Fig. 4d).

### Effects of intra-articular senescent microenvironment (IASM) on the MSCs

**Fig. 5:**
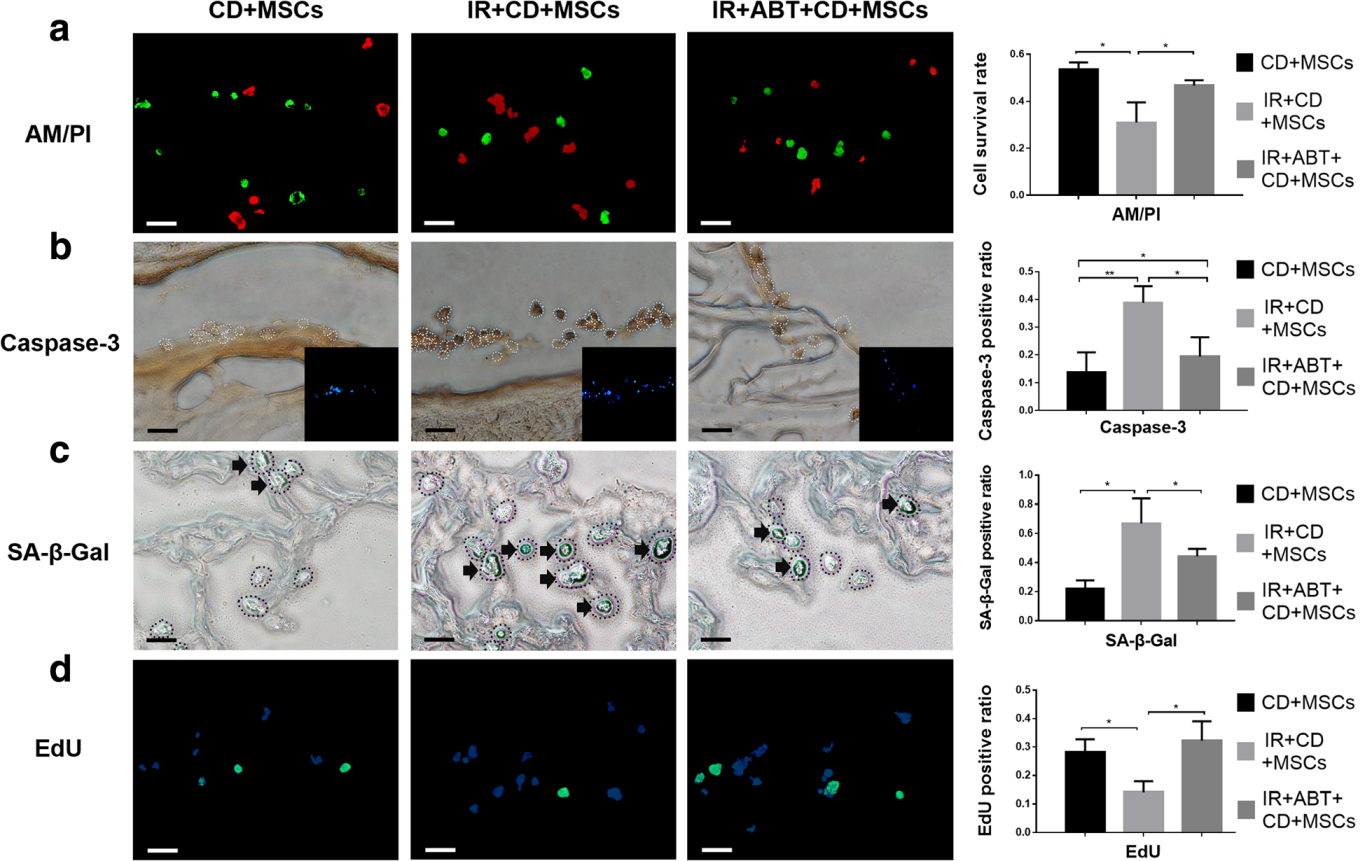
The effect of IASM on viability, apoptosis, senescence, and proliferation of MSCs in rat joint. **a**, **b**, **c**, **d** The Calcein-AM (green)/PI (red) (**a**), Caspase-3 (brown) (**b**), SA-β-Gal (green) (**c**), and EdU (green) (**d**) staining of MSCs in gel scaffold in cartilage defect 1 week after transplantation. Nucleus were stained by Hoechst 33342 (blue). Bars = 50 μm. Positive cells were calculated respectively (right, *n* = 3). *P < 0.05, **P < 0.01

We evaluated the effects of IR-induced intra-articular senescent microenvironment (IASM) on the viability, apoptosis, senescence, and proliferation of transplanted BMSCs at 1 week. Calcein-AM/PI staining revealed that 53% BMSCs in agarose gel survived 1 week after transplantation. IASM reduced this viability to 31%, while intra-articular ABT pretreatment improved it to 47% (Fig. 5a).

Thirteen percent BMSCs in the non-IR group were positively stained for the caspase-3. IASM enhanced this to 39%, and intra-articular ABT reduced it to 18% (Fig. 5b).

In the non-IR group, 22% BMSCs exhibited positive SA-β-Gal staining. IASM significantly promoted cellular senescence of transplanted BMSCs. Sixty-seven percent MSCs in IR-treated joint were SA-β-Gal positive. These effects were attenuated by ABT-263 (Fig. 5c).

Likewise, EdU staining revealed that IASM inhibited the proliferation of transplanted BMSCs. Twenty-eight percent of MSCs were positive for EdU in the non-IR joints. IASM reduced this ratio to 14%, while ABT improved it to 32% (Fig. 5d).

### Effects of IASM on the cartilage regeneration

**Fig. 6:**
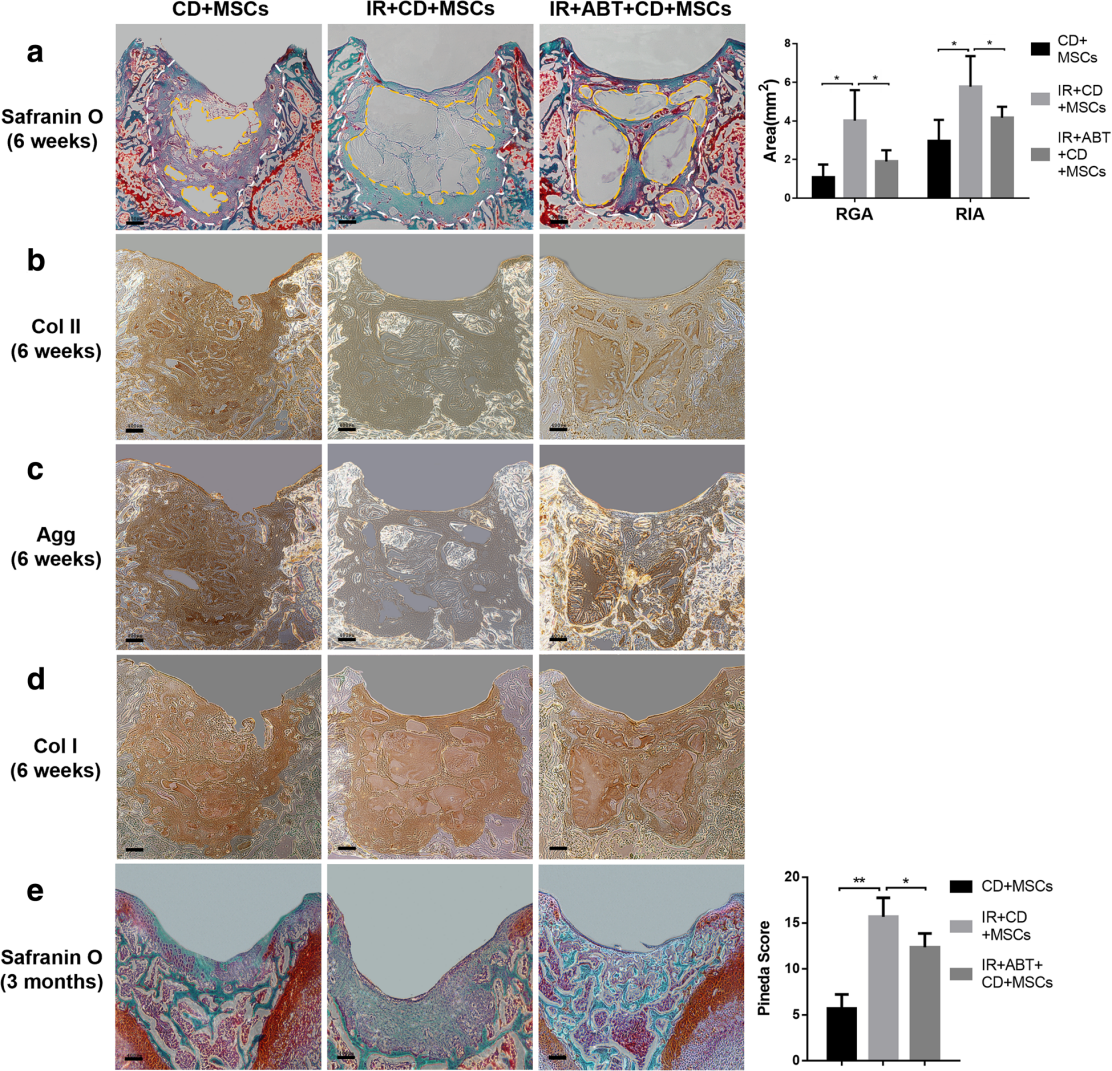
The effect of IASM on BMSCs cartilage regeneration. **a** Safranin O/fast green staining of cartilage repair 6 weeks after BMSCs implantation. The residual immature area (RIA, white dashed line) and residual gel area (RGA, yellow dashed line) were calculated (right, *n* = 3). Bars = 400 μm. **b**–**d** The immunohistochemistry staining for Col II (**b**), Agg (**c**), and Col I (**d**) of cartilage repair 6 weeks after BMSCs implantation. Bars = 400 μm. **e** Safranin O/fast green staining of cartilage repair 3 months after BMSCs implantation. Bars = 400 μm. The Pineda score of cartilage regeneration was evaluated (right, *n* = 3). *P < 0.05, **P < 0.01

We evaluated the effect of IASM on cartilage regeneration at 6 and 12 weeks. Overall, grafts stayed in situ throughout the experiment with no graft detachment observed. No obvious features of joint degeneration were noted. Neocartilage in all groups integrated well to surrounding tissues. Regenerated tissue and residual gel were found to be interlaced at 6 weeks. Compared with the non-IR group, IASM generated a cartilage repair with significant larger residual immature area (RIA) and residual gel area (RGA) at 6 weeks. Furthermore, the regenerated tissues in IASM revealed negative staining of safranin O. ABT partially improved these results (Fig. 6a). The Agg and Col II IHC staining exhibited similar results. The regenerated tissues in the non-IR group exhibited strong positive staining for Agg and Col II, while IASM weakened these staining. ABT provided a partial improvement (Fig. 6b, c). Col I was observed in all three groups, and neither IASM nor ABT had obvious effect on Col I expression (Fig. 6d). At 3 months, the non-IR group was found to have satisfying cartilage repair, although regions of aberrant tissue were present. However, IASM resulted in the collapse of reparative tissue and abnormal cellular morphology. ABT was able to induce cartilage repair that consisted of ossified tissue without cartilage morphology (Fig. 6e).

## Discussion

Senescent cells (SnCs) are often present in tissues afflicted by age-related diseases, such as OA [3]. The heterogeneous secretory profile of SnCs, termed SASP, creates a microenvironment that affects the stem cells around SnCs. In our study, SnChos stimulated MSC chondrogenic markers expression in short-term co-culture, while prolonged SnChos exposure mainly resulted in maintenance of cellular stemness and inhibition of differentiation (Fig. 4a, c). In animal experiments, 6 weeks IASM exposure resulted in the reduction of Col II, Agg expression, and safranin O staining in neocartilage (Fig. 6a-c). The exact mechanism of this bidirectional effect is presently unknown. Senescent microenvironments affect cell phenotype in a complex manner. The transient presence of SnCs was reported to be beneficial for wound healing through PDGF-A-induced myofibroblast differentiation [14]. We believe the temporary SnCs exposure is a beneficial physiological mechanism for tissue repair by facilitating stem cell recruitment and differentiation, given the abundance of chemokines and growth factors in SASP [6]. However, chronic existence of SnCs appears harmful to tissue regeneration by regulating cell stemness. Recently, the model of “senescence-stem lock” had been given attention [6]. This mechanism is believed to underlie the impairment of SnCs to tissue regeneration. A number of SASP factors, such as IL-6, appear to facilitate neighboring cell pluripotency [15, 16]. The persistent existence of SnCs continuously releasing these SASP factors are thought to ‘lock’ these cells in a pluripotent state, therefore hampering the appropriate differentiation of stem cells required for tissue repair. Similar to our results, hematopoietic stem cells derived from the senescent bone marrow exhibited defects in multilineage differentiation [17, 18]. The accumulated SnCs in fat with aging inhibited the adipogenic markers expression in differentiating adipose stem cells [19]. Weilner et al. found that circulating microvesicles derived from senescent endothelial cells inhibited osteogenic differentiation of MSCs and accelerated osteoporosis [20]. These appear to explain the results that, comparing with traumatic cartilage defect, the cell-based therapies for even early-stage OA exhibited poor phenotype of regenerated tissue [21].

However, not all studies regard the senescent microenvironment as an inhibitor of stem cell differentiation. Infante and Rodríguez found that the secretome of SnCs was able to stimulate osteogenic differentiation of human MSCs [22]. Moreover, the role of IL6, a key SASP factor, in chondrogenic differentiation of MSCs is still controversial. IL-6 was demonstrated to promote the chondrogenic differentiation of MSCs in a dose-dependent manner [23]. But in the other study, IL-6 inhibited this chondrogenic differentiation [24]. In our study, the fibrous tissues were also observed in regenerated tissues of non-IR joints. Neither IASM nor ABT treatment had obvious effect on Col I expression (Fig. 6d). It seems that other undesired factors, instead of senescent microenvironment, caused the fibrosis commonly seen during cartilage regeneration.

The decline in stem cell quantity is another issue affecting tissue regeneration. Proliferation restriction, accompanied by apoptosis, reduced the cell number after transplantation. We found that Chos appeared to intrinsically stimulated MSCs proliferation (Fig. 1c). In contrast, results revealed that SnChos markedly suppressed MSC proliferation (Figs. 1c and 5d), inducing apoptosis (Figs. 2a and 5b) and senescence (Figs. 3a and 5c). In fact, chondrocytes with complete senescent phenotype were less than 20% in the SnChos group at 7 days (Fig. 3b), while majority of chondrocytes were relatively normal. That means merely a fraction of SnChos was sufficient to reverse the positive effect of Chos and lead to senescence and apoptosis of stem cells. This appears to provide support for the debridement of cartilage lesion margin, which contains abundant SnChos, before stem cell implantation. Cellular senescence occurred in cells surrounding SnCs. Studies revealed that SnCs induced stem cell senescence and inhibited their colony formation [7, 17, 25–27]. For example, it was recently demonstrated that the conditioned medium (CM) from Senescent MSCs triggered deceleration of cell proliferation and induced a senescent phenotype of normal MSCs. The senescence inducing pathway appeared to be transmitted through p53/p21/Rb and p16/MAPKAPK-2/Rb signaling [26]. Ritschka et al. believed that the effect of SnCs on cell proliferation was also bidirectional [7]. A short-term (2 days) and mild SASP exposure induced cell stemness and stimulated proliferation of keratinocytes, while prolonged (6 days) existence of SASP caused cellular senescence. However, we failed to observe this bidirectional effect in our co-culture. SnChos appear to have a major negative effect on MSC proliferation.

The anti-senescence therapy for age-related diseases was the subject of intense research [17, 27]. Jeon et al. recently found that clearance of SnCs from degenerated joints attenuated the progression of OA [4]. We attempted to explore whether removing of SnChos prior to BMSC transplantation could improve MSC behavior. Both in vitro and in vivo studies revealed that clearance of SnChos using anti-senescence agent ABT-263 attenuated those deleterious effects on BMSCs, including induction of cellular apoptosis (Figs. 2a and 5b) and senescence (Figs. 3a and 5c) and inhibition of chondrogenesis (Figs. 4a, c and 6). This provides a strategy to facilitate tissue regeneration of stem cells by improving intra-articular senescent microenvironment. However, except the semigenetic clearance of SnCs in animal models, none of the anti-senescence agents, including ABT-263, are completely specific toward SnCs presently [6]. The development of new agents with superior specificity and lower off-target toxicity are required. It was found that stem cells also possessed anti-senescence property. But the effect of BMSCs to SnChos was rarely studied. We found that BMSCs reduced SA-β-Gal-positive cells and stimulated cell proliferation in the SnChos group (Figs. 1d and 3b). Exosomes, CM, and secretomes derived from various stem cells were reported to reverse the senescent phenotype of human dermal fibroblasts induced by irradiation, as evidenced by changes in SA-β-gal staining, MMP secretion, proliferation, and matrix protein generation [28–30]. Wang et al. revealed that the secretome from human fetal MSCs could ameliorate replicative senescence of culture-expanded human adult MSCs [31]. It was unclear whether these effects were a result of direct reversal of the senescent phenotype or from elimination of the SnChos subgroup, just like the mechanism of ABT-263. We found that BMSCs revealed similar pattern of influence with ABT on SnChos proliferation, senescence, and apoptosis. ABT pretreatment further reinforced the effects of BMSCs on SnChos (Figs. 1d, 2b and 3b). Therefore, it is more logical that BMSCs eliminated SnChos by inducing cell apoptosis, thus improving the local senescent context and removing suppression of proliferation to the surrounding relative normal chondrocytes. However, study revealed that the CM from adipose stem cells was able to reduce SnChos heterochromatic foci formation and p53 acetylation [32], which was critical in cell apoptosis. Therefore, it was also reasonable that BMSCs had reversed the senescent phenotype of SnChos. SnCs promote a low-level chronic local inflammation through SASP factors such as inflammatory cytokines and MMPs, resulting in age-related decline in organ function. This was termed as “inflammaging,” as exemplified by the SnChos in OA cartilage [33]. In our study, MSCs revealed a bidirectional effect on SnChos inflammaging. Although prolonged interaction revealed an obvious anti-inflammaging effect of MSCs, there was also a transient but dramatic increase of IL and MMP expression in SnChos within 7 days of co-culture with BMSCs (Fig. 4b). ABT pretreatment significantly attenuated this transient promotive effect of BMSCs (Fig. 4b) and enhanced the Col II production in SnChos group while BMSCs alone had no effect (Fig. 4d). These seem to imply that, in addition to elimination of SnChos, BMSCs had unique effects on SnChos in cartilage repair and degeneration. But the underlying mechanism has yet to be clarified. It is possible that this effect is beneficial for cartilage remodeling at the interface between damaged cartilage and MSC grafts. But, the Chos and MSCs were cultured in pattern of monolayer in this study. This might impair the phenotype of cells and affect the outcomes [34]. Further study is required.

## Conclusions

The interactions between SnCs and stem cells are presently an area of active study. In this study, we found that SnChos imposed comprehensive effects on MSCs in co-culture, including cellular senescence, apoptosis, stemness, and differentiation. Meanwhile, MSCs mainly revealed an anti-senescence effect, perhaps by inducing the apoptosis of SnChos. The IASM impaired the survival, proliferation, and appropriate differentiation of grafted MSCs in rat joint and suppressed the cartilage repair. These interactions are likely to underlie the mechanisms that hamper the MSC performance in cartilage regeneration. In addition, this study provided a rationale for the exploration of usage of anti-senescence agents in regenerative medicine.

## Additional file

Additional file 1: **Figure S1.** The identification of BMSCs chondrocytes. **a-e** BMSCs isolated from rats were identified by cell morphology (**a**), osteogenesis (**b**, Alizarin Red staining), adipogenesis (**c**, Oil Red O staining), chondrogenesis (d, Alcian Blue staining) differentiation and surface biomarkers (**e**). **f** Primary chondrocytes isolated from rats were identified using type II collagen immunocytochemistry. Bars = 100 μm. (PNG 1437 kb)

## Abbreviations

BMSCs: Bone marrow mesenchymal stem cells
CD: Cartilage defect
Chos: Chondrocytes (non-senescent)
IASM: Intra-articular senescent microenvironment
OA: Osteoarthritis
SASP: Senescence-associated secretory phenotype
SnChos: Senescent chondrocytes
