# Supplementary figures and images for: Intraarticular senescent chondrocytes impair the cartilage regeneration capacity of mesenchymal stem cells
Source: Stem Cell Res Ther. 2019 Mar 12;10:86. doi: 10.1186/s13287-019-1193-1 (PMC6416972; doi:10.1186/s13287-019-1193-1)

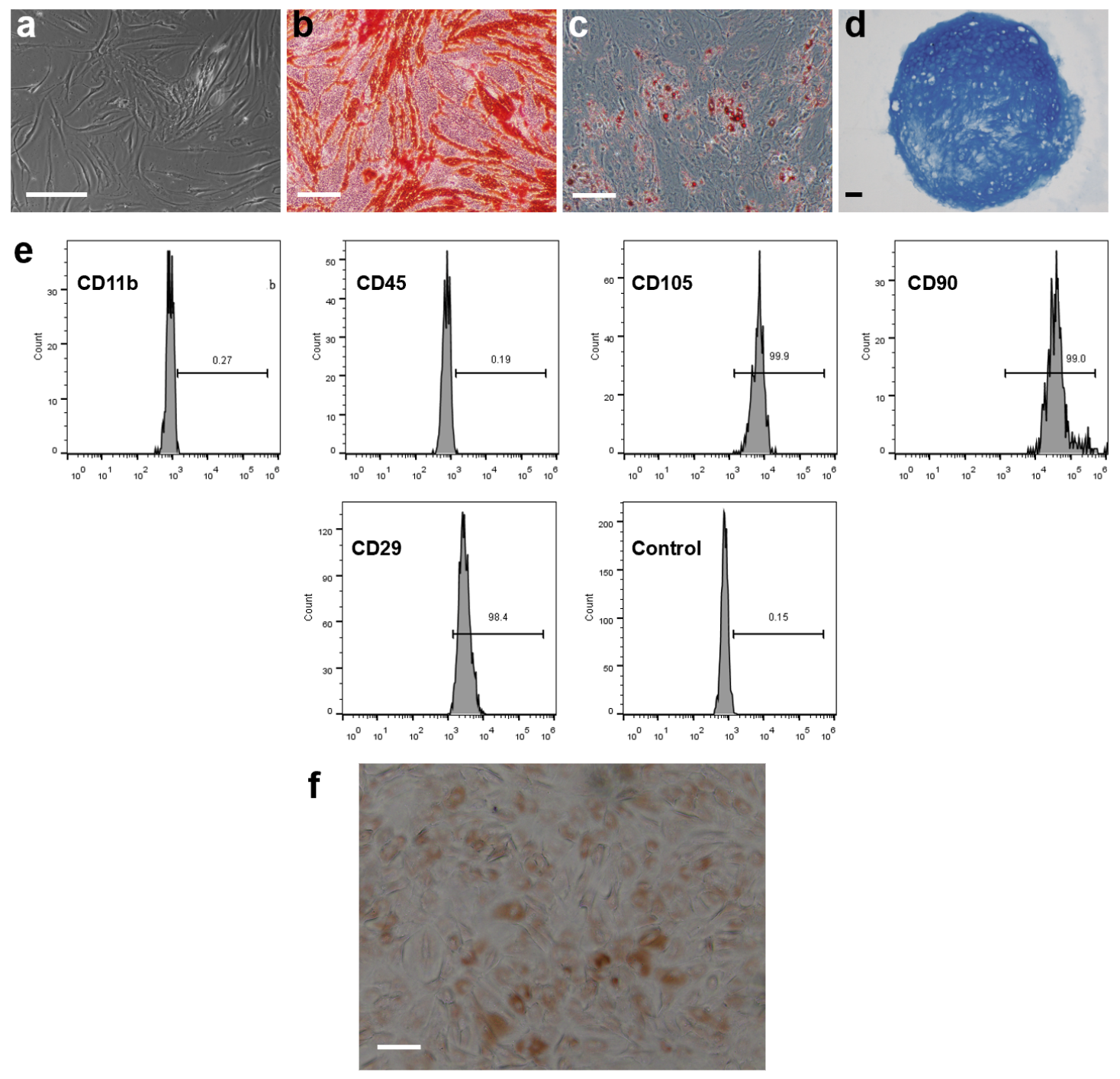

Supplement: Supplementary file 1 — Figure S1. The identification of BMSCs chondrocytes. a-e BMSCs isolated from rats were identified by cell morphology (a), osteogenesis (b, Alizarin Red staining), adipogenesis (c, Oil Red O staining), chondrogenesis (d, Alcian Blue staining) differentiation and surface biomarkers (e). f Primary chondrocytes isolated from rats were identified using type II collagen immunocytochemistry. Bars = 100 μm. (PNG 1437 kb) [file 13287_2019_1193_MOESM1_ESM.png]
